# FGFR1 Overexpression Induces Cancer Cell Stemness and Enhanced Akt/Erk-ER Signaling to Promote Palbociclib Resistance in Luminal A Breast Cancer Cells

**DOI:** 10.3390/cells10113008

**Published:** 2021-11-04

**Authors:** Qiong Cheng, Zhikun Ma, Yujie Shi, Amanda B. Parris, Lingfei Kong, Xiaohe Yang

**Affiliations:** 1 Department of Pathology, Henan Provincial People’s Hospital, People’s Hospital of Zhengzhou University, Zhengzhou 450003, China; 10050084@vip.henu.edu.cn (Q.C.); yujie-shi@zzu.edu.cn (Y.S.); 2 Department of Biological and Biomedical Sciences, Julius L. Chambers Biomedical/Biotechnology Research Institute, North Carolina Central University, Kannapolis, NC 28081, USA; maz2@vcu.edu (Z.M.); ablack18@nccu.edu (A.B.P.); 3 Lineberger Cancer Center, University of North Carolina at Chapel Hill, Chapel Hill, NC 27514, USA

**Keywords:** FGFR1, palbociclib, CDK4/6 inhibitor resistance, breast cancer, cancer stem cells, cyclins/CDKs

## Abstract

Resistance to CDK4/6 inhibitors (CDKis) is emerging as a clinical challenge. Identification of the factors contributing to CDKi resistance, with mechanistic insight, is of pivotal significance. Recent studies linked aberrant FGFR signaling to CDKi resistance. However, detailed mechanisms are less clear. Based on control and FGFR1 overexpressing luminal A cell line models, we demonstrated that FGFR1 overexpression rendered the cells resistant to palbociclib. FGFR1 overexpression abolished palbociclib-mediated cell cycle arrest, as well as the attenuated palbociclib-induced inhibition of G1/S transition regulators (pRb, E2F1, and cyclin D3) and factors that promote G2/M transition (cyclin B1, cdc2/CDK1, and cdc25). Importantly, FGFR1-induced palbociclib resistance was associated with promotion of cancer cell stemness and the upregulation of Wnt/β-catenin signaling. We found that palbociclib may function as an ER agonist in MCF-7/FGFR1 cells. Upregulation of the ER-mediated transcription in MCF-7/FGFR1 cells was associated with ERα phosphorylation and enhanced receptor tyrosine kinase signaling. The combination of palbociclib with FGFR-targeting AZD4547 resulted in remarkable synergistic effects on MCF-7/FGFR1 cells, especially for the inhibition of cancer cell stemness. Our findings of FGFR1-induced palbociclib resistance, promotion of cancer stem cells and associated molecular changes advance our mechanistic understanding of CDKi resistance, which will facilitate the development of strategies targeting CDKi resistance in breast cancer treatment.

## 1. Introduction

Cell cycle deregulation with aberrant activation of cyclin dependent kinases (CDKs) is a hallmark of cancer [1]. CDK4/6 inhibitors (CDKis) are a class of novel anti-cancer agents targeting the G1/S transition of the cell cycle. Since 2015, three CDKis, including palbociclib, ribociclib, and abemaciclib, have been approved by the FDA for patients with hormonal receptor-positive and human epidermal growth factor receptor 2-negative (HR+/HER2−) advanced breast cancer [2]. In combination with endocrine therapy, CDKi treatment has markedly improved progression-free and overall survival, which is a paradigm-shifting advancement in breast cancer therapy [2,3]. Despite the high efficacy of CDKis, the development of drug resistance, either acquired resistance after continued treatment or de novo resistance (in certain subgroups of cancer), is emerging as a serious challenge that may significantly reduce the clinical benefits of CDKis [4,5,6,7]. Therefore, understanding the mechanism of CDKI resistance is of pivotal importance.

Data from both preclinical models and clinical reports indicate that the development of resistance to CDKis is inevitable [8,9,10,11]. Certain subgroups of ER+/Her2- breast cancer are resistant to CDKi de novo [12,13]. To address this critical clinical problem, substantial efforts have been made to tackle CDKi resistance issues, in order to further the development of novel therapeutic strategies to improve clinical benefits [6,14]. A number of factors associated with CDKi resistance have been identified via various approaches, such as ORF kinome screening of cells treated with ribociclib and fulvestrant [10], as well as whole-exome sequencing of breast cancer tissues in paired settings of pre-treatment and post-resistance to ER-targeted therapy [15]. Mechanisms of CDKi resistance, associated with cell cycle regulation, include loss of Rb, amplification/overexpression of cyclin E1, p16, E2F, CDK6, and other cell cycle regulators, which promotes the cancer cells to bypass CDK4/6 inhibition [5,8,16,17]. Mechanisms associated with the deregulation of growth factor receptors and downstream signaling in PI3K/AKT/mTOR pathways, such as mutations in PI3K catalytic subunit (PIK3CA) and FGFR1/2 amplification/overexpression, are another major group of factors that contribute to CDKi resistance [18,19,20]. Despite these advances, understanding the molecular mechanisms of individual factors contributing to CDKi resistance and identifying novel biomarkers monitoring CDKi responses are in urgent need, in order to improve CDKi efficacy in breast cancer treatment [2].

Fibroblast growth factor receptor 1 (FGFR1) is a member of the FGFR family, which is a group of receptor tyrosine kinases (RTKs) that are activated by binding to their cognate ligands, the FGFs [21]. It plays a critical role in physiological mammary development and tissue homeostasis [22]. FGFR1 amplification and/or overexpression is frequently detected in breast and other cancers [21,23]. In particular, we previously reported that FGFR1 overexpression, which is more common in the luminal A and B subtypes of breast cancer, is an adverse outcome indicator for luminal A breast cancer, which is characterized by ER+/Her2- [24]. FGFR amplification/overexpression has also been associated with tumor development and resistance to endocrine therapy [25,26,27]. In addition, increasing reports indicate that FGFR signaling is involved in the regulation of mammary and cancer stem cells (CSCs) [28,29,30]. FGFR-deregulated CSCs are also associated with resistance to chemotherapy agents [30]. Recently, aberrant FGFR signaling was identified as a critical factor contributing to CDKi resistance [10]. In addition to FGFR1, a genome-wide functional screening showed that two activating FGFR2 mutations (M538I and N550K) were associated with resistance to palbociclib and/or fulvestrant in endocrine-resistant ER+ metastatic breast cancer [15]. FGFR1/2 amplifications or mutations were observed in 41% (14/34) of the patients who progressed after CDKi therapy in the MONALEESA-2 trial [4,10]. In a study using NSCLC models, acquired palbociclib resistance was associated with ERK-activated mTOR, which was driven in part by FGFR1 signaling, resulting from extracellular secretion of FGF ligands [31]. Overexpression of FGFR1 in MCF-7 or T47D cells promoted resistance to CDKi. The combination of fulvestrant and palbociclib with FGFR-targeting lucitanib enhanced tumor inhibition of FGFR1-overexpressing cancer cells [10]. While these data underscore the significance of FGFR deregulation in endocrine-CDKi combined therapy, the detailed mechanisms of FGFR1 overexpression-induced CDKi resistance require further investigation. Although the FGFR1 signaling-mediated promotion of CSCs was associated with resistance to different therapeutic agents in previous studies [30,32], whether and how FGFR1 overexpression regulates CSCs in CDKi resistance has not been reported.

In this study, we aimed to understand molecular mechanisms associated with FGFR1 overexpression-induced palbociclib resistance. Using isogenic cell line models, we demonstrate that FGFR1 overexpression in MCF-7 and T47D cells renders them resistant to palbociclib. In particular, we found that the resistance involves the FGFR1-induced promotion of CSC stemness, in addition to its abolition of palbociclib-induced G1/S arrest. These were accompanied by the FGFR1-induced activation of cyclins/CDKs, beyond the CDK4/6-pRb-E2F1 axis, and upregulation of ER-RTK crosstalk and Wnt signaling. Our findings will advance our understanding of FGFR1-mediated palbociclib resistance for developing strategies to overcome CDKi resistance.

## 2. Materials and Methods

### 2.1. Reagents and Antibodies

Palbociclib, AZD4547, fulvestrant/ICI-182,780, LY294002, and PD98059 were purchased from LC Laboratories (Woburn, MA, USA); bFGF was purchased from StemCell Technologies (Vancouver, BC, USA). FGFR1 (9740), p-Akt (Ser473) (4060), p-Erk1/2 (Thr202/Tyr204) (9101), p-pRb (Ser807/811) (8516), pRb (9309), cdc2 (9116), cdc25 (4688), Cyclin B1 (12231), Cyclin D1 (2978), p-ERα (Ser118) (2511), p-ERα (Ser167) (5587), c-Myc (5605), TCF1 (2203), p-FRS2 (Tyr196) (3864), LRP6 (3395), and p-LRP6 (Ser1490) (2568) primary antibodies were purchased from Cell Signaling Technologies (Danvers, MA, USA). Akt1 (sc-5298), Erk2 (sc-1647), ERα (sc-8002), E2F1 (sc-251), Cyclin D3 (sc-182), CDK4 (sc-260), and β-actin (sc-47778) primary antibodies were purchased from Santa Cruz Biotechnology (Santa Cruz, CA, USA). Active β-catenin (05-665) antibody was purchased from EMD Millipore (Billerica, CA, USA).

### 2.2. Cell Culture

MCF-7 and T47D breast cancer cell lines were purchased from the American Type Culture Collection (ATCC) (Manassas, VA, USA). MCF-7/Control (MCF-7/C), MCF-7/FGFR1, T47D/Control (T47D/C), and T47D/FGFR1 were stable sublines established in our lab, as described previously [27]. The cells were maintained in DMEM/F-12 culture medium supplemented with 10% fetal bovine serum (FBS), penicillin (100 μg/mL), and streptomycin (100 μg/mL) in a humidified incubator with 5% CO_2_ at 37 °C.

### 2.3. Cell Proliferation Assay

Cell Proliferation Kit II (XTT, Roche, CA, USA) was used in the evaluation of cell proliferation/survival. The cells were seeded into 96-well plates (1 × 10^3^ cells/well) for 24 h and then treated with palbociclib or AZD4547 (or in combination) at the indicated concentrations for 5 days. XTT reagent was then incubated with the cells for four hours at 37 °C. The colorimetric absorbance was measured with an ELISA microplate reader (BioTek; Winooski, VT, USA) at 450 nm. Six parallel replicates were analyzed for each experimental sample. Representative data from three sets of repeats were presented.

### 2.4. Clonogenic Assays

Cells were plated (1 × 10^3^ cells/well) in 6-well plates for 24 h, followed by treatment with palbociclib or AZD4547 at indicated concentrations for 14 days, with a media change at regular time intervals. Then, the colonies were fixed with 70% methanol and stained with 0.5% crystal violet. Colonies were counted using ImageJ software and images captured with the FluorChemE imager (Cell Biosciences; Santa Clara, CA, USA). Results, based on triplicate data, were statistically analyzed with student *t*-test. Representative data from three sets of repeats were presented.

### 2.5. Cell Cycle Analysis with Flow Cytometry

Cells were treated with palbociclib for 24 h. Appropriately treated cells were collected by trypsinization and fixed in 70% ethanol overnight at −20 °C. The fixed cells were washed in PBS and incubated for 30 min at 37 °C in 0.05% Triton X-100/PBS, RNase A (100 μg/mL), and propidium iodide (PI; 50 μg/mL). Cells in different cell cycle phases were analyzed using a Guava easyCyte 8 flow cytometer (Millipore; Billerica, MA, USA). The percentage of the cells, in individual phases, was analyzed with ModFit software [33]. Representative data from three sets of repeats were presented.

### 2.6. Transwell Migration and Invasion Assay

The invasion potential of tumor cells was evaluated using the 24-well BD Growth Factor Reduced Corning Matrigel Invasion Chamber, as in our previous studies [27]. Briefly, cell suspensions (2 × 10^4^/well) in serum-free DMEM/-F12 medium, supplied with palbociclib, AZD4547, or in combination, were inoculated into the upper chamber pre-coated with Matrigel, while the lower chamber was filled with DMEM/F-12 containing 10% FBS. The cells were incubated for 36 h at 37 °C, and the cells that migrated/invaded through the Transwell membrane were fixed with methanol, stained with 0.1% crystal violet, and counted under a light microscope at 200× magnification. An average of five visual fields were examined and each assay was performed in triplicate.

### 2.7. Tumorsphere Assays

Cells were seeded (1000 cells/well) in ultra-low attachment 24-well plates (Corning). The cells treated with indicated doses of palbociclib, AZD4547, or in combination, were incubated in DMEM/F-12 medium, supplemented with 10 μg/mL insulin (Sigma; St. Louis, MO, USA), 1 μg/mL hydrocortisone (Sigma), 1× B27 (Thermo Fisher Scientific; Waltham, MA, USA), 20 ng/mL EGF (Stemcell Technologies; Vancouver, BC, USA), 20 ng/mL bFGF (Stemcell Technologies), and 4 μg/mL heparin (Stemcell Technologies) for 7 days, in order to access primary sphere formation [33]. The spheres between 40–120 μM in diameter were counted and imaged. Single-cell suspensions, harvested from the primary spheres using trypsinization, were then replated for evaluation of secondary sphere formation using conditions same to primary spheres, followed by sphere counting and imaging. Primary and secondary sphere formation, based on triplicate settings, were analyzed with Student’s *t*-test.

### 2.8. Western Blot Analysis

Cell lysates were prepared from treated cells, as in our previous report [33]. Protein concentrations were quantified using a BCA Protein Assay kit (Thermo Scientific; Rockford, IL, USA), and equal amounts of proteins (50 µg) were separated on 8–15% SDS-PAGE gels and transferred onto nitrocellulose membranes. Membranes were blocked in 5% milk in TBST for 2 h at room temperature, followed by incubation with specific primary antibodies at 4 °C overnight. Membranes were then washed in a TBST buffer and incubated in horseradish peroxidase (HRP) secondary antibodies for 2 h at room temperature. After final washes in TBST buffer, protein bands were detected using enhanced chemiluminescence (ECL) reagents (Thermo Scientific) and imaged using the FluorChemE imager. Representative images of the three repeats were presented. Since we detected a number of markers from each set of lysate/condition, multiple membranes were probed, with a close monitoring of protein concentrations and loading control using β-actin.

### 2.9. Luciferase Assay

Cells were seeded in 12-well plates and incubated overnight. The cells were transfected with plasmids encoding ERE-firefly luciferase and control Renilla luciferase with X-tremeGENE 9 (Roche; Indianapolis, IN, USA), as described previously [34]. The cells were then serum-starved for 48 h, followed by treatment with E2 (10 nM), palbociclib (5 µM) alone or in combination in phenol red-free DMEM/F-12 medium with 5% Charcoal Stripped FBS for 24 h. The luciferase activity was measured using Luciferin Detection Reagent (Promega; Madison, WI, USA) and a Modulus single tube reader (Turner BioSystems; Sunnyvale, CA, USA). Normalized firefly luciferase activity of each group from both sublines was compared to the MCF-7/C cells for the calculation of relative ERE-mediated transcriptional activities.

### 2.10. Quantitative RT-PCR (qRT-PCR)

Total RNA was isolated from cells with Trizol (Thermo Fisher Scientific), as per the manufacturer’s instructions. Purified RNA (1 μg) was used to synthesize cDNA using the iScript cDNA synthesis kit (Bio-Rad; Hercules, CA, USA). The qPCR assay was carried out on a Bio-Rad CFX96 system using SYBR green mastermix. The primer sequences are in Supplementary Table S1. Relative mRNA levels were quantified based on the cycle threshold (Ct) values of the tested genes, as normalized to the control, the *GAPDH* gene [27].

### 2.11. Drug Synergy Analysis

The synergetic effect between palbociclib and AZD4547 was evaluated using XTT assays. The CI values were calculated using the Chou–Talalay method, with the CompuSyn software (ComboSyn, Inc.; Paramus, NJ, USA) [35]. CI < 1, CI = 1, and CI > 1 indicate synergistic, additive, and antagonistic effects, respectively.

### 2.12. Statistical Analysis

Data analysis was performed using software from Prism 7 (GraphPad; La Jolla, CA, USA) and data were expressed as means ± SE. IC_50_s of MCF-7 and MCF-7/FGFR1 cells and statistical differences were analyzed with nonlinear regression using Prism 7. Data from clonogenic, tumorsphere, invasion and luciferase assays, and qPCR were analyzed with two-tailed Student’s *t* tests. The *p* values of ≤0.05 are considered statistically significant.

## 3. Results

### 3.1. FGFR1 Overexpression Significantly Reduces Palbociclib-Induced Inhibition of Proliferation and Invasion of MCF-7 Cells

**Figure 1:**
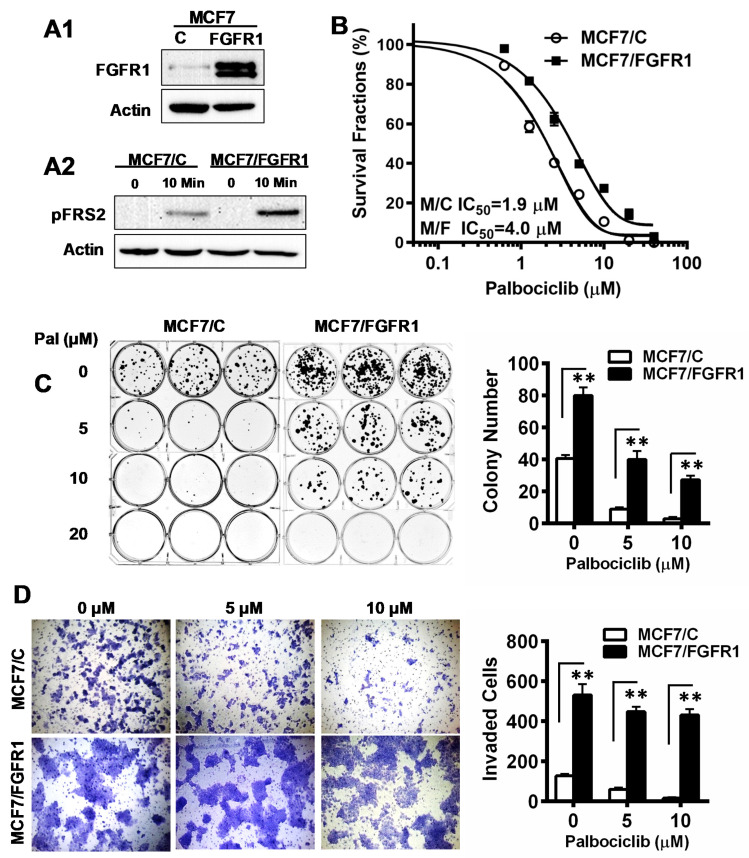
FGFR1 overexpression in MCF-7 cells induces resistance to palbociclib-mediated inhibition of proliferation and invasion. (**A1**) FGFR1 levels in MCF-7/C and MCF-7/FGFR1 cells. (**A2**) FGFR1-induced activation of FRS2 in MCF-7/C and MCF-7/FGFR1 cells. The cells were starved with serum-free media for 48 h and then treated with bFGF (100 ng/mL) for 10 min, followed by Western blot analysis. (**B**) MCF-7/C and MCF-7/FGFR1 cells were treated with palbociclib (0–40 µM) for 5 days, followed by an XTT assay. IC_50_ values were analyzed with Prism 7. (**C**) Clonogenic assays of drug-treated MCF-7/C and MCF-7/FGFR1 cells. The cells were treated with palbociclib (0, 5, 10 μM) for 2 weeks. The colonies were stained with crystal violet and quantified. (**D**) Transwell invasion assay. The paired cell lines were treated with palbociclib (0, 5, 10 μM) for 30 h in invasion chambers. The cells that invaded through the transwell were stained, counted, and analyzed. Data in (**C**,**D**) were statistically analyzed with Student’s *t*-test (** *p* < 0.01).

MCF-7 is an ER+/Her2- breast cancer cell line, frequently used to test the efficacy of CDKis. To study the impact of FGFR1 deregulation on therapeutic resistance, we established stable control (MCF-7/C) and FGFR1-overexpressing (MCF-7/FGFR1) sublines (Figure 1A1). To show that FGFR1 overexpression was functional, we demonstrated that MCF-7/FGFR1 cells were more sensitive to bFGF stimulation than MCF-7/C cells (Figure 1A2). The effect of FGFR1 overexpression on palbociclib resistance was then assessed with XTT assays (Figure 1B). When MCF-7/C and MCF-7/FGFR1 cells were treated with palbociclib, at concentrations ranging from 0 to 40 μM for 5 days, the survival fractions in FGFR1-overexpressing cells were consistently higher than that of the corresponding control cells under the same conditions. The IC_50_ and 95% confidence interval (95% CI) for MCF-7/C and MCF-7/FGFR1 were 1.9 μM (95% CI: 1.81 to 2.04 μM) and 4.0 μM (95% CI: 3.79 to 4.26 μM), respectively (*p* < 0.01) (Figure 1B). In parallel, we examined the effect of palbociclib on clonogenic cell survival of MCF-7/C and MCF-7/FGFR1 cells. In contrast to sensitive inhibition of colony formation in MCF-7/C cells, which show fewer colonies in the 5 and 10 μM groups, clonogenic formation efficiencies in palbociclib-treated MCF-7/FGFR1 cells, under the same conditions, were significantly higher than that of MCF-7 cells (Figure 1C). The relative inhibition in drug-treated cells, over the corresponding untreated control, also showed that palbociclib-induced inhibition in MCF-7/FGFR1 cells was attenuated (Figure S1). Data from both assays indicate that FGFR1 overexpression in MCF-7 cells significantly negated the palbociclib-induced inhibition of proliferation in these cells. We next compared the effect of palbociclib on cell migration/invasion between MCF-7/C and MCF-7/FGFR1 cells using invasion chamber assays. As in Figure 1D and Figure S2, palbociclib-induced a profound inhibition of cell invasion in MCF-7/C cells. On the contrary, both the number of invaded cells and the ratio of drug-treated groups over untreated control in MCF-7/FGFR1 cells were higher than MCF-7/C cells under the same conditions, indicating that palbociclib-mediated inhibition of cell invasion and migration were significantly attenuated in these cells.

### 3.2. FGFR1 Abolishes Palbociclib-Induced Cell Cycle Arrest through Activation of Cell Cycle Regulators beyond Cyclin D-CDK4/6–pRb Pathway

**Figure 2:**
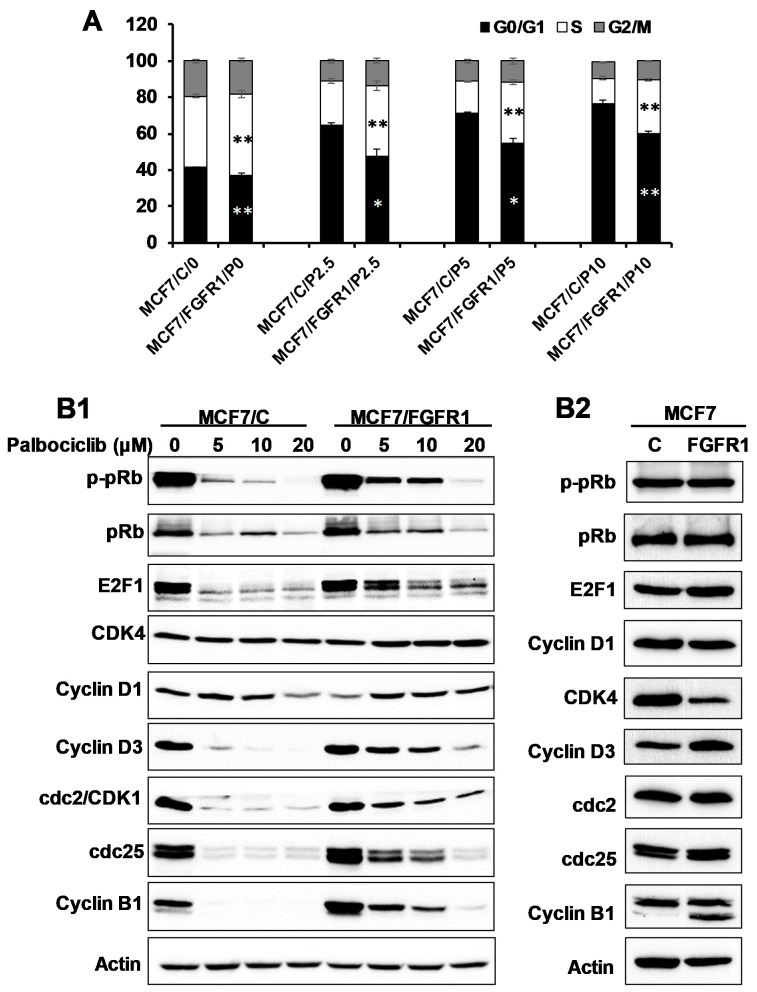
Effect of FGFR1 overexpression on palbociclib-induced inhibition of cell cycle progression and cell cycle regulators. (**A**) MCF-7/C and MCF-7/FGFR1 cells were treated with palbociclib (0, 2.5, 5, 10 μM) for 24 h, followed by cell cycle analysis. Cells in different phases were analyzed with ModFit software. (** *p <* 0.01, * *p* < 0.05 vs. corresponding phases of control cells). Data from triplicate samples were reported. (**B1**) Western blot detection of key cell cycle regulators regulated by palbociclib. MCF-7/C and MCF-7/FGFR1 cells were treated with palbociclib, at the indicated concentrations, for 48 h, followed by Western blot analysis of specified markers. (**B2**) Protein levels of key markers in (**B1**) in MCF-7/C and MCF-7/FGFR1 cells in the absence of palbociclib.

The primary target of CDKis is the cyclin D-CDK4/6–pRb pathway that regulates G1/S transition of the cell cycle [14]. Consistent with the proliferation data above, cell cycle analysis showed that palbociclib-induced cell cycle arrest in MCF-7/C cells, as indicated by increased G0/G1-phase cells and decreased S phase cells (Figure 2A). In MCF-7/FGFR1 cells, however, palbociclib-induced G0/G1 arrest was attenuated, as indicated by a significant increase in S phase cells. The data suggest that FGFR1 overexpression counteracts palbociclib-induced cell cycle arrest. To understand the underlying mechanism, we next examined the effect of FGFR1 overexpression on key cell cycle regulators, including cyclin D-CDK4/6–pRb pathway and additional cyclins/CDKs. We found that palbociclib significantly downregulated phospho-pRb (p-pRb), E2F1, pRb, and cyclin D3 in MCF-7/C cells. These downregulations, however, were markedly decreased in MCF-7/FGFR1 cells, indicating the molecular basis of FGFR1 overexpression-mediated attenuation of G1/S transition inhibition by palbociclib. Moreover, we found the protein levels of CDK4 and cyclin D1 in palbociclib-treated MCF-7/C cells were not significantly downregulated. Their levels were only modestly upregulated in drug-treated MCF-7/FGFR1 cells. Although palbociclib is primarily a CDKi, we found that the protein levels of cdc2/CDK1, cdc25, and cyclin B1 were sensitively downregulated in palbociclib-treated MCF-7/C cells, which was also significantly attenuated in MCF-7/FGFR1 cells (Figure 2B1). Since the differences between the two cell lines were more significant in palbociclib-treated groups, as compared to non-drug-treated cells (Figure 2B2), our results suggest that FGFR1 overexpression may have more functional impact on cell cycle progression, in the presence of palbociclib-induced cell cycle arrest. Taken together, these results demonstrate that FGFR1 overexpression-induced attenuation of palbociclib-induced cell cycle arrest is not only mediated by counteracting palbociclib’s inhibition of cyclin-CDK-pRb/E2F network but is also associated with activation of non-G1/S cyclins and CDKs, which may contribute to palbociclib resistance.

### 3.3. FGFR1 Overexpression-Induced Palbociclib Resistance Is Associated with Enhanced CSC Stemness and Upregulation of Wnt Signaling

**Figure 3:**
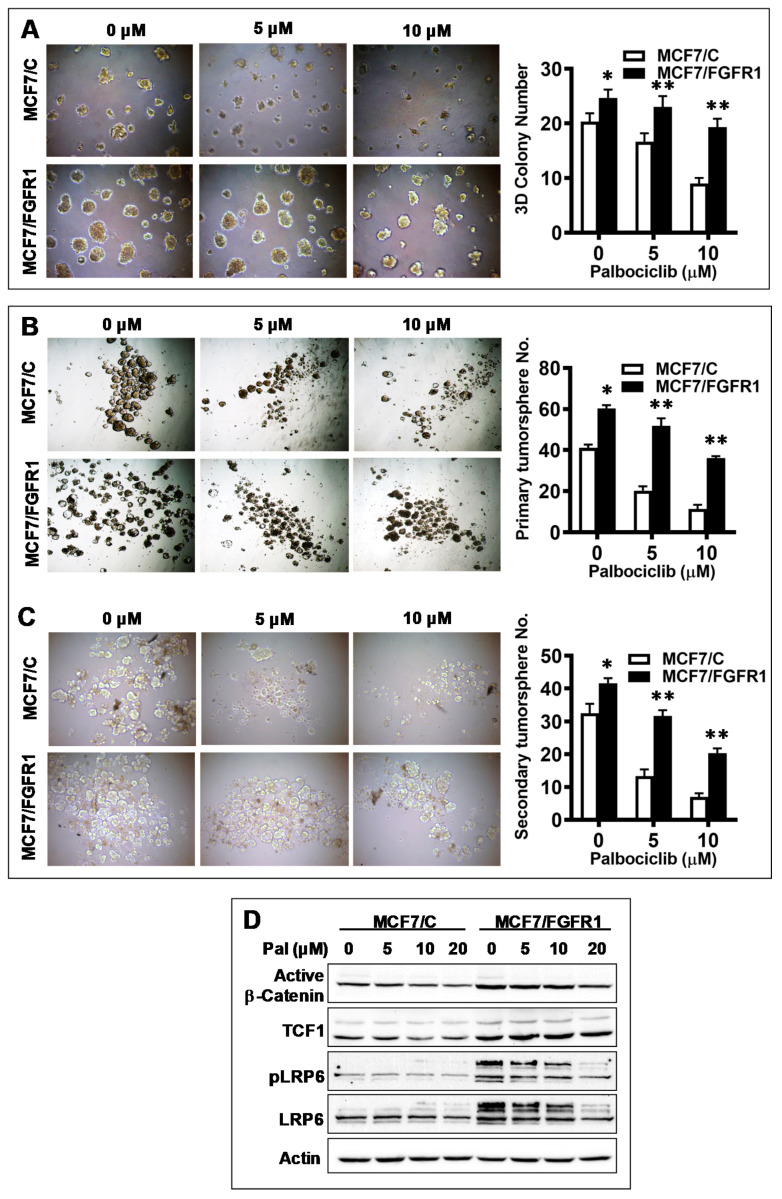
FGFR1 overexpression induces CSC stemness in palbociclib-treated MCF-7 cells. (**A**) Evaluation of anchorage-independent growth in palbociclib-treated MCF-7/C and MCF-7/FGFR1 cells with 3D culture. The cells cultured in matrigel were treated with palbociclib (0, 5, 10 μM) for 7 days, followed by colony imaging and analysis. (**B**,**C**) Tumorsphere assays of palbociclib-treated MCF-7/C and MCF-7/FGFR1 cells. The cells were plated in ultra-low attachment plates and treated with palbociclib (0, 5, 10 μM) for 7 days in triplicate. Primary tumorspheres (**B**) were imaged and counted for analysis. Single-cell suspensions, harvested from corresponding primary spheres, were then replated for secondary sphere formation (**C**) and analysis. (**D**) Western blot detection of markers in Wnt signaling. MCF-7/C and MCF-7/FGFR1 cells were treated with palbociclib at indicated concentrations for 48 h, followed by Western blot analysis of specified markers. Data in (**A**–**C**) were analyzed by Student’s *t*-test (** *p* < 0.01, * *p* < 0.05 vs. corresponding control cells).

CSC promotion has been connected with FGFR1-associated therapeutic resistance [30], but its role in CDKi resistance has not been studied. To this end, we examined CSC stemness of palbociclib-treated MCF-7/C and MCF-7/FGFR1 cells. Results from 3D culture assays, which reflect cancer cell stemness in anchorage-independent growth, indicate that palbociclib induced a significant inhibition of 3D colonies in MCF-7/C cells, whereas 3D colonies in palbociclib-treated MCF-7/FGFR1 cells were greater than the control cells under the same conditions in number, colony size (Figure 3A), and relative inhibition (Figure S3). In the tumorsphere assays, a useful tool to analyze the self-renewal ability of CSCs, we found that the efficiency of sphere formation, especially the secondary sphere formation that indicates self-renewal potential of CSCs, in MCF-7/C cells was significantly inhibited by palbociclib. In contrast, palbociclib-mediated inhibition of tumorsphere formation in MCF-7/FGFR1 cells was significantly attenuated (Figure 3B,C and Figure S4). Data from both 3D culture and tumorsphere assays provide direct evidence showing that promoting CSC stemness is involved in FGFR1 overexpression-induced palbociclib resistance. Since Wnt/β-catenin signaling is one of the major pathways involved in CSC regulation [36], we examined key markers of this pathway in palbociclib-treated MCF-7/C and MCF-7/FGFR1 cells. We found that protein levels of active β-catenin, along with downstream factor TCF1, were upregulated in MCF-7/FGFR1 cells (Figure 3D). We also found that protein levels of total and phosphorylated LRP6, which is upstream of β-catenin [37], were upregulated concomitantly. The data suggest that activation of Wnt/β-catenin pathway in MCF-7/FGFR1 cells may partially explain the enhanced stemness and resistance of FGFR1-overexpressing cells treated with palbociclib.

### 3.4. FGFR1 Activates ER-Mediated Transcription through Induction of ERα Phosphorylation by Activated Akt and ERK1/2 in MCF-7/FGFR1 Cells

**Figure 4:**
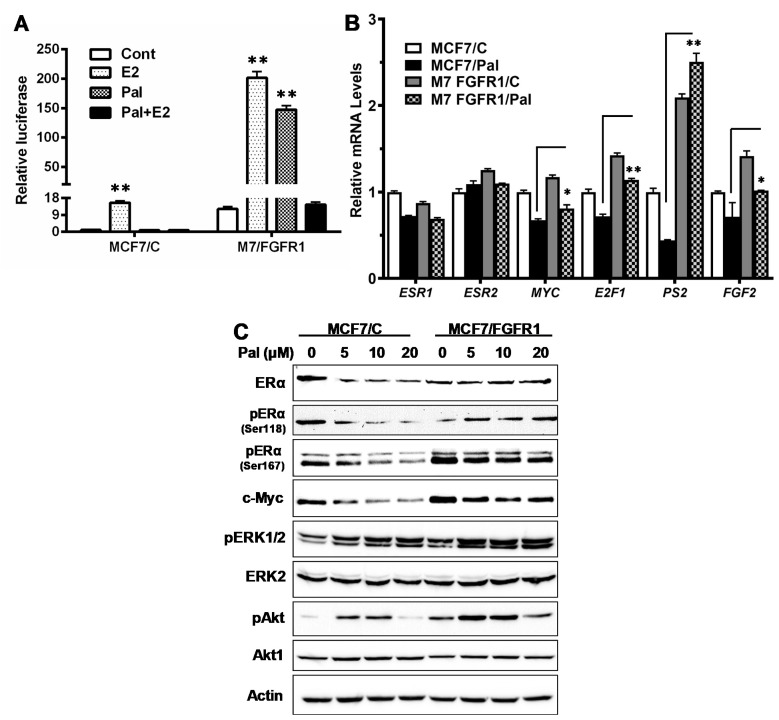
Effect of FGFR1 overexpression on ER-mediated transcription and ERα phosphorylation. (**A**) Luciferase reporter assay of ERE-mediated transcription in MCF-7/C and MCF-7/FGFR1 cells. The cells were transfected with plasmids encoding an ERE-firefly luciferase and control Renilla luciferase, followed by treatment with E2 (10 nM) and palbociclib (5 µM), alone or in combination, for 24 h. The relative luciferase activity of each cell line is normalized and compared to the control of MCF-7/C cells. (**B**) qPCR analysis of mRNA levels of pertinent ER target genes (*ESR1/ERα, ESR2/ERβ, MYC, E2F1, PS2,* and *FGF2*) in MCF-7/C and MCF-7/FGFR1 cells treated with palbociclib (0, 5 µM) for 24 h. (**C**) Western blot detection of ERα phosphorylation in context with c-Myc expression and the phosphorylation of Akt and ERK1/2. MCF-7/C and MCF-7/FGFR1 cells were treated with palbociclib, at the indicated concentrations, for 48 h, followed by Western blot analysis of specified markers. Values in (**A**,**B**) based on triplicate samples were analyzed with Student’s *t*-test (* *p* < 0.05; ** *p* < 0.01).

Activation of the FGFR pathway may induce ER signaling and resistance to endocrine therapy [26]. A previous study showed that combination of palbociclib with ER-targeting fulvestrant and FGFR-targeting Lucitanib resulted in enhanced inhibition of FGFR1-overexpressing breast cancer cells [10], but the response of ER signaling in the treated cells was not thoroughly analyzed. We, therefore, examined the effect of FGFR1 overexpression on ER signaling, in context with palbociclib treatment. We first assessed ER-mediated transcriptional activity in the control and FGFR1-overexpressing cells using the ERE-luciferase reporter system. In MCF-7/C cells, 10 nM E2 induced a 15-fold increase in ERE activity; and palbociclib treatment was able to keep E2-induced ERE activity under check. In contrast, the basal ERE activity in the MCF-7/FGFR1 cells was ~12 fold of the control cells; and E2 induced a ~200-fold of ERE activity in these cells. Intriguingly, ERE activity in MCF-7/FGFR1 cells was induced ~150-fold by palbociclib alone and ~15 fold by E2 in combination with palbociclib (Figure 4A). These results not only indicate that FGFR1 overexpression strongly upregulates ER signaling in palbociclib-treated cells, but also suggests that palbociclib could function as an ER agonist under certain conditions in FGFR1-overexpressing breast cancer cells.

We next used qPCR to examine the relative mRNA levels of a few ER target genes involved in ER signaling (*ESR1/ERα, ESR2/ERβ,* and *PS2*), cell cycle (*E2F1* and *MYC*), and FGFR ligand *FGF2* in MCF-7/C and MCF-7/FGFR1 cells. As in Figure 4B, the mRNA levels of these genes were inhibited by palbociclib, to various degrees, in MCF-7/C cells. In MCF-7/FGFR1 cells, however, the transcription of most of these genes were upregulated, even in the presence of palbociclib. Specifically, the upregulation of PS2 gene was most significant, whereas the transcription of ESR1 and ESR2 in palbociclib-treated cells was not increased, possibly due to a net result of multi-pathway regulation. Nevertheless, the mRNA levels of E2F1, MYC, and FGF2 in palbociclib-treated MCF-7/FGFR1 cells were greater than the drug-treated MCF-7/C cells. The data suggest that FGFR1 overexpression-induced upregulation of ER-mediated transcription is a critical factor contributing to palbociclib resistance in FGFR1-overexpressing breast cancer cells.

Activation of ER signaling could be induced by increased ER expression and/or phosphorylation by MAPK/ERK1/2 and PI3K/Akt signaling. Phosphorylated ERα (pERα)/Ser118 and pERα/Ser167 are known targets of ERK1/2 and Akt-mediated phosphorylation [38]. Our analysis indicates that the total protein levels of ERα were not upregulated in MCF-7/FGFR1 cells, as compared to MCF-7/C cells (Figure 4C). However, both pERα/Ser118 and pERα/Ser167, along with c-Myc, a classical ER target, were markedly increased in MCF-7/FGFR1 cells, which was accompanied by the increase in p-ERK1/2 and p-Akt in these cells. These results suggest that FGFR1 induces ER signaling, mainly through activation of ERα by phosphorylation. Of note, we noticed that palbociclib alone induced a modest activation of ERK1/2 and Akt in MCF-7/C cells at lower concentrations. In context with the data in Figure 4A,B, FGFR1-induced ER phosphorylation through the activation of ERK1/2 and Akt signaling appears to be the major mechanism that induces enhanced activation of ER signaling in palbociclib-treated MCF-7/FGFR1 cells. This may be coupled with FGF2 upregulation and the ER-mediated expression of growth factors/ligands that activate Akt/Erk signaling [38] to enhance FGFR1 overexpression-induced ER-RTK crosstalk.

### 3.5. Combination with FGFR-Targeting Agent AZD4547 Enhances Palbociclib-Induced Inhibition of FGFR1 Overexpressing Cancer Cells

**Figure 5:**
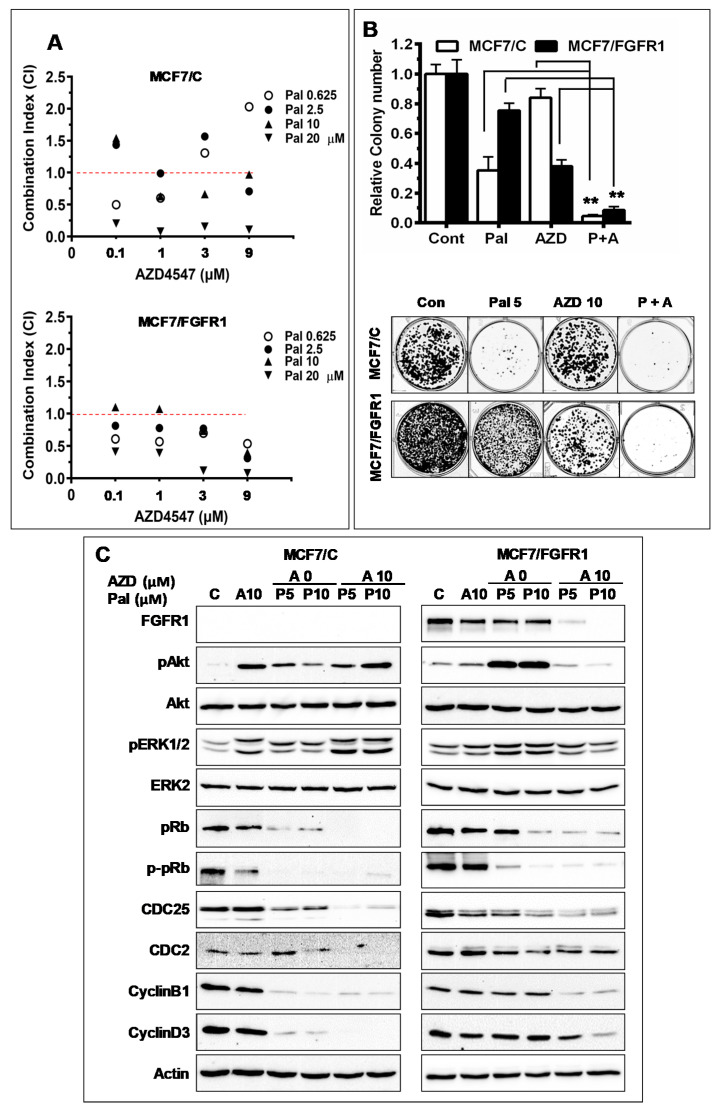
Effect of AZD4547 combination on palbociclib-induced inhibition of proliferation and signaling in MCF-7/C and MCF-7/FGFR1 cells. (**A**) Combination with AZD4547 synergizes palbociclib-induced inhibition of cell proliferation. MCF-7/C and MCF-7/FGFR1 cells were treated with palbociclib (0, 0.625, 1.25, 2.5, 5, 10, 20 µM) ± AZD4547 (0, 0.1, 0.3, 1, 3, 9 µM) for 5 days, followed by XTT assays. The survival fractions for each cell line were normalized to the control. The combination index (CI) of palbociclib and AZD4547 from the XTT assays for each cell line was calculated using CompuSyn software. The spots with CI < 1.0 indicate synergistic effect. (**B**) Effect of AZD4547 and palbociclib combination on clonogenesis. The MCF-7/C and MCF-7/FGFR1 cells inoculated for clonogenic assays were treated with palbociclib (5 μM) and AZD4547 (10 μM), alone or in combination, for 2 weeks. The colonies were stained with crystal violet for imaging and analysis with Student’s *t*-test (** *p* < 0.01). (**C**) Western blot detection of key markers in RTK signaling and cell cycle regulation in palbociclib-treated cells. MCF-7/C and MCF-7/FGFR1 cells were treated with palbociclib (0, 5 μM, 10 μM) and AZD4547 (10 μM), alone or in combination, for 48 h, followed by Western blot analysis of specified markers.

To overcome FGFR1 overexpression-induced palbociclib resistance, we tested the effect of combination of palbociclib with FGFR targeted inhibitor AZD4547 on cell proliferation and clonogenesis of MCF-7/C and MCF-7/FGFR1 cells. The cells were treated with palbociclib (0, 0.625, 2.5, 10, 20 µM) in the absence or presence of AZD4547 (0, 0.1, 1, 3, 9 µM), followed by survival fraction assessments to determine whether AZD4547 is synergistic with palbociclib. The combination index (CI) of the two drugs in each cell line was analyzed with the CompuSyn software [35]. As shown in Figure 5A, AZD4547 synergistically enhanced palbociclib-induced growth inhibition, as indicated by the points with CI ≤ 1.0, in the treated cells.

We further demonstrated the synergistic effect of AZD4547 on palbociclib-mediated inhibition with colony-formation assays (Figure 5B). When the cells were treated with 5 µM palbociclib and 10 µM AZD4547 alone or in combination, the groups with either drug alone displayed substantial number of colonies. In contrast, combination of the drugs resulted in remarkable inhibition of colony formation. The synergistic effect was more evident in FGFR1-overexpressing cells than the control cells.

To understand the molecular basis of the synergistic effect, we examined the relative protein levels of key markers associated with cell cycle regulation and RTK signaling in the paired cell lines treated with palbociclib alone or in combination with AZD4547 (Figure 5C). The results clearly demonstrated that combination of AZD4547 markedly enhanced palbociclib-mediated inhibition of p-pRb, cyclin B1, cyclin D3, and cdc25 in MCF-7/FGFR1 cells. Importantly, the combination offsets Akt phosphorylation/activation induced by palbociclib alone. Of note, the decrease of FGFR1 in AZD4547 treated MCF-7/FGFR1 cells was possibly caused by AZD4547-induced FGFR degradation [39]. Collectively, corresponding changes of these critical regulators may partially explain the synergistic phenotypes observed above.

### 3.6. Combination with AZD4547 Abrogates FGFR1 Overexpression-Associated Promotion of Invasion and CSC Stemness in Palbociclib-Treated Cells

**Figure 6:**
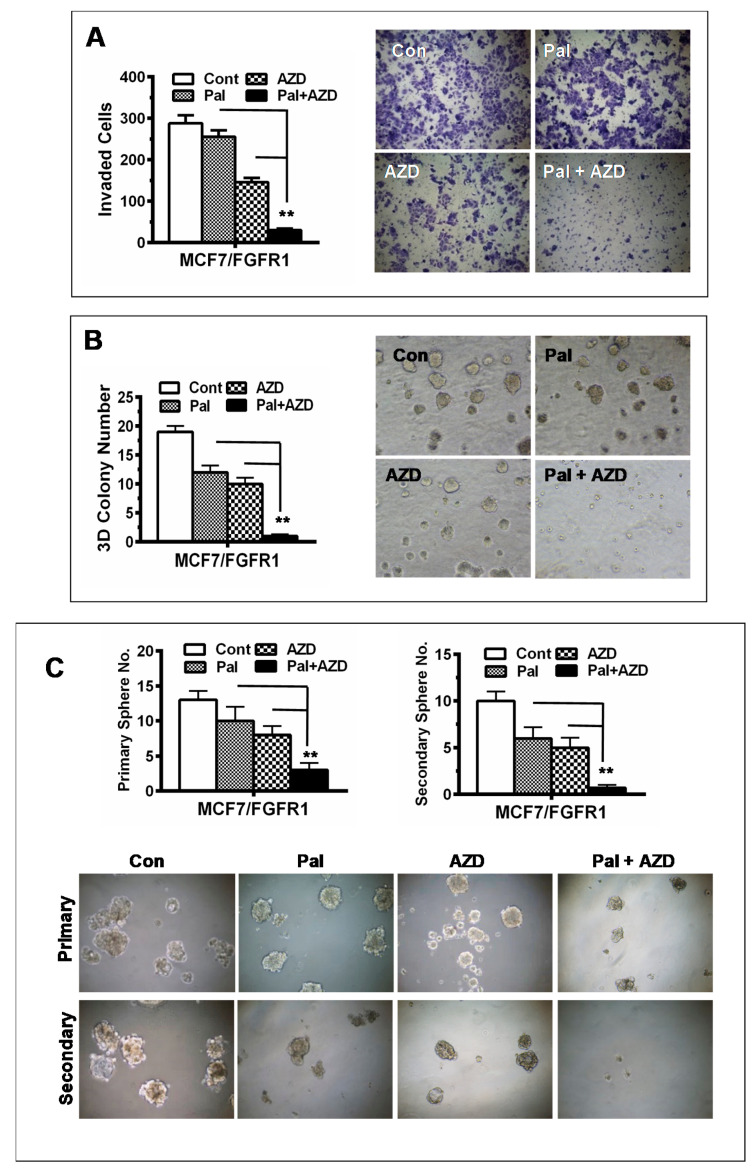
Combination with AZD4547 synergistically enhances palbociclib-induced inhibition of cell invasion and CSC stemness. (**A**) Transwell invasion assays. MCF-7/FGFR1 cells were treated with palbociclib (5 μM) and AZD4547 (5 μM) alone or in combination for 30 h in invasion chambers. The invaded cells were stained, counted, and analyzed. (**B**) 3D culture of MCF-7/FGFR1 cells with different treatments. The cells were treated with palbociclib (5 μM) and AZD4547 (5 μM) alone or in combination in matrigel 3D culture for 7 days, followed by colony imaging and counting. The numbers of colonies in triplicate were analyzed. (**C**) Effect of drug combination on tumorsphere formation. MCF-7/FGFR1 cells were plated in ultra-low attachment plates and treated with 5 μM AZD4547 and 5 μM palbociclib, alone or in combination, in triplicate. Primary tumorspheres were counted and imaged after 7 days. Then single cell suspensions harvested form primary spheres were replated for secondary sphere formation for another 7 days. The spheres at each harvest were imaged and counted. Values in each set of experiments were analyzed with Student’s *t*-test (** *p* < 0.01 vs. single drug treatment).

Since palbociclib resistance is associated with enhanced invasion and stemness in MCF-7/FGFR1 cells (Figure 1 and Figure 3), we examined the effect of palbociclib in combination with AZD4547 on the invasion and stemness of MCF-7/FGFR1 cells. As in Figure 6A, each drug alone induced partial inhibition of cell migration/invasion. In contrast, combination of the two drugs essentially blocked MCF-7/FGFR1 cell invasion in the Transwell invasion assays. In the determination of palbociclib-AZD4547 combination-induced inhibition of anchorage-independent growth, we found that the combination efficiently abrogated the 3D colony formation potential of MCF-7/FGFR1 cells (Figure 6B). We next tested the effect of the combination on tumorsphere formation and self-renewal. As in Figure 6C, combination of palbociclib with AZD4547 significantly inhibited sphere formation of MCF-7/FGFR1 cells, as compared to individual drug alone. In particular, the formation of secondary spheres, which represent CSC self-renewal potential, in the cells treated with both drugs was abolished. These data support the strategy of combination of palbociclib with FGFR-targeting agent in this subtype of breast cancer and demonstrate the effectiveness of blocking both CDK4/6 and FGFR signaling pathways in targeting the CSC stemness involved in the drug resistance.

### 3.7. The Effect of Inhibitor Targeting PI3K/Akt, MAPK/Erk, ERα, and LRP6 Pathways on FGFR1 Overexpression-Induced Signal Transduction

**Figure 7:**
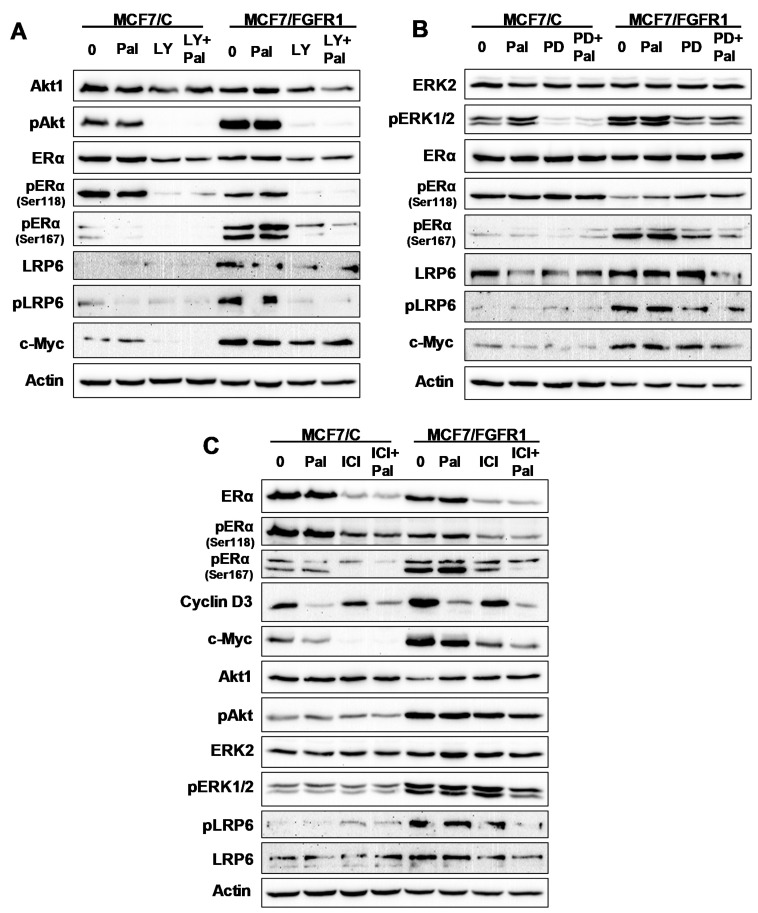
Effect of inhibitor targeting PI3K/Akt, MAPK/Erk, ERα, and LRP6 pathways on FGFR1 overexpression-induced signal transduction. PI3K/Akt targeting LY294002, MAPK/Erk targeting PD98059 and ER targeting fulvestrant/ICI 182,780 (ICI) were used for the tests. MCF-7/C and MCF-7/FGFR1 cells were treated with palbociclib (0, 5 μM) and each specific inhibitor alone or in combination for 24 h, followed by Western blot analysis of specified markers. (**A**) The paired cell lines were treated with palbociclib and/or LY294002 (20 μM); (**B**) the paired cell lines were treated with palbociclib and/or PD98059 (20 μM); (**C**) the paired cell lines were treated with palbociclib and/or ICI (100 nM). Each panel was probed with the target of the specific inhibitor and other key markers of RTK, ER, and LRP6 signaling.

The above experiments suggested that FGFR1 overexpression-induced palbociclib resistance involves the activation and interactions of PI3K/Akt, MAPK/Erk and ER signaling pathways. However, the mechanistic insight of each pathway is not clear. We, therefore, used specific inhibitors targeting individual pathways, including PI3K/Akt targeting LY294002, MAPK/Erk targeting PD98059, and ER targeting fulvestrant/ICI 182,780 (ICI), to address this question (Figure 7). Consistent with above data, palbociclib alone induced little or less inhibition of the key markers of these pathways, as compared to MCF-7/C cells. Combination of palbociclib with LY294002 not only inhibited Akt activation/phosphorylation but also resulted in the inhibition of pERα/Ser167, pERα/Ser118, and pLRP6, indicating the interactions between Akt and ER signaling, as well as Akt with LRP6 (Figure 7A). Combination of palbociclib with PD98059 led to enhanced inhibition of ERK1/2, pERα/Ser118, pERα/Ser167, pLRP6, and cMyc (Figure 7B). Combination of palbociclib with fulvestrant/ICI induced enhanced inhibition of total and phospho-ERα and c-Myc, pAkt, pERK1/2 and pLRP6 (Figure 7C). Although the detailed interactions may require further characterization, these data suggest that the cross talking network among Akt/Erk-ERα-LRP6 pathways is involved in FGFR1-overexpressing mediated palbociclib resistance.

### 3.8. Overexpression of FGFR1 Induces Palbociclib Resistance in T47D Cells

**Figure 8:**
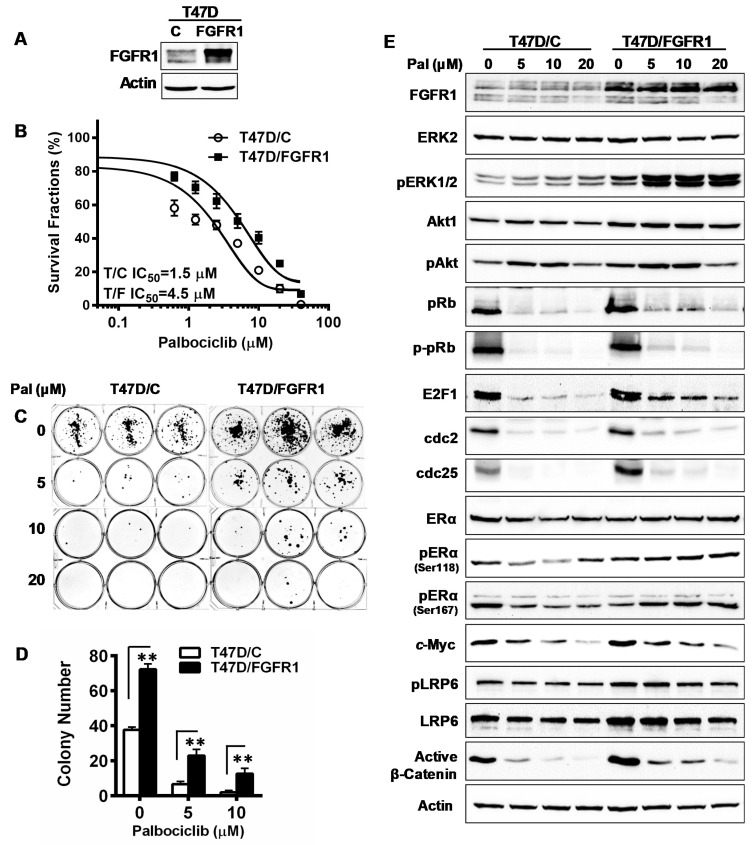
FGFR1 overexpression in T47D cells induces resistance to palbociclib-mediated inhibition, with modified signaling in RTK, ER, and LRP6 pathways. (**A**) FGFR1 protein levels in T47D/C and T47D/FGFR1 cells. (**B**) T47D/C and T47D/FGFR1 cells were treated with palbociclib (0–40 µM) for 5 days, followed by an XTT assay. Survival fractions and IC_50_ values based on data of 8 replicates were analyzed with Prism 7. (**C**,**D**) Clonogenic assays of T47D/C and T47D/FGFR1 cells treated with palbociclib. The cells, in triplicate, were treated with palbociclib (0, 5, 10 μM) for 2 weeks. The colonies were stained with crystal violet and quantified. The data were statistically analyzed with Student’s *t*-test (** *p* < 0.01). (**E**) Western blot detection of indicated markers of relevant pathways in T47D/C and T47D/FGFR1 cells treated with palbociclib. The cells were treated at indicated concentrations for 48 h, followed by Western blot analysis of specified markers.

To verify the findings from the MCF-7 cell line model, we examined the effect of FGFR1 overexpression on palbociclib response using another luminal A breast cancer cell line, T47D. We first developed T47D/C and T47D/FGFR1 sublines and then tested them with palbociclib treatment (Figure 8). Results from XTT assays showed that T47D/FGFR1 cells were more resistant than T47D/C cells to palbociclib, as indicated by the IC_50_ of each subline (Figure 8B). Data from clonogenic assays showed that FGFR1 overexpression attenuated palbociclib-induced inhibition of colony formation in T47D cells (T47D/FGFR1 vs. T47D/C), as indicated by both colony numbers and the relative inhibition normalized with untreated control (Figure 8C,D and Figure S5). We next examined the key markers of cell cycle regulation, RTK signaling, ER, and LRP6/Wnt pathways in drug-treated T47D/C and T47D/FGFR1 cells. As shown in Figure 8E, the differences of protein levels of the indicated markers between the paired cell lines were in similar patterns to that of MCF-7/C and MCF-7/FGFR1 cells. In T47D/C cells, palbociclib induced a marked downregulation of pRb, E2F1, cdc2, cdc25, pERα/Ser167, c-Myc, and active β-catenin. In contrast, these changes were decreased in T47D/FGFR1 cells. Consistent data between MCF-7 and T47D cell line models provide further support of the FGFR1 overexpression-induced palbociclib resistance.

## 4. Discussion

With increasing use of CDKis in breast cancer treatment, the development of resistance is becoming an important clinical problem [14]. Recently, several lines of evidence linked aberrant FGFR signaling to CDKi resistance, including data from the ORF kinome screening and whole genome sequencing of breast cancer tissues involving CDKi treatments [10,15]. The combination of the FGFR-targeting agent with CDKi and endocrine therapy improved the inhibition of PDX tumors [20]. Although these reports provided a proof of concept of FGFR deregulation-associated CDKi resistance, detailed mechanisms of the cellular and molecular changes associated with the resistance are less clear. In the current study, we used luminal A breast cancer cell line models to demonstrate FGFR1-induced palbociclib resistance with mechanistic insight. Compared to the previous studies with multifactorial involvement, the marked differences between palbociclib-treated MCF-7/C and MCF-7/FGFR1 cells, as well as T47D/C and T47D/FGFR1 cells, revealed specific cellular changes that are associated with FGFR1 overexpression and palbociclib resistance. We found that FGFR1 overexpression induced counteractions on the palbociclib-mediated inhibition of proliferation and invasion, which was reflected not only in direct readouts but also in relative ratios of drug-treated over untreated cells of each line (Figures S1–S5). These phenotypic changes were associated with the concurrent activation of cyclin/CDK-pRb/E2F signaling and enhanced ER-RTK crosstalk. In particular, enhanced CSC stemness and Wnt signaling, detected in palbociclib-treated MCF-7/FGFR1 cells, as well as changes in cell cycle blockage, highlight the role of CSC deregulation in FGFR-induced CDKi resistance, providing clues to further mechanistic studies. Given the shared pathways downstream of the FGFs/FGFRs signaling cascade, our findings may also shed light on CDKi resistance, associated with other forms of FGFR deregulation. In addition to FGFR1 overexpression, aberrant FGFR signaling in breast cancer may also include the amplification of FGFR1 and FGFR2, activation/mutations of FGFR2, increased secretion of FGF2, and other ligands from tumor microenvironment [4,10,40]. Therefore, our results will have a broader impact on CDKi resistance associated with deregulation of FGF/FGFR signaling.

How FGFR deregulation confers CDKi resistance is an essential question. It is known that the fundamental mechanism of CDKi-mediated anti-tumor activity is that inhibition of CDK4/6 decreases CDK4/6/cyclin D-induced phosphorylation of pRb, which results in the inhibition of the E2F-mediated transcription of genes involved in G1/S transition [14]. Consistently, we found that palbociclib-treated MCF-7/FGFR1 cells had a higher percentage of S phase cells and lower percentage of G0/G1 cells than the control cells, indicating attenuated inhibition of G1/S transition. This is further supported by reduced inhibition of phosphorylated pRb and E2F1 levels in palbociclib-treated MCF-7/FGFR1 cells. These results provide direct evidence that FGFR1 overexpression counteracts palbociclib-induced inhibition of CDK4/6-pRb signaling. Regarding the mechanisms of FGFR1 deregulation-associated CDKi resistance, a previous study proposed that cyclin D1 may be the major mediator of FGFR1-induced CDKi resistance [10]. This was supported by the frequent (~30%) co-amplification of FGFR1 and cyclin D1/CCND1, as well as a cyclin D1 upregulation in the tumor tissues, that was examined [10]. However, FGFR1 overexpression-induced cyclin D1 upregulation was not prominent in our cell line models. Our data, from repeated verification, showed that protein levels of cyclin D1 and CDK4 were not markedly upregulated before or after palbociclib treatment (Figure 2 and Figure 8). In contrast, the levels of p-pRb, E2F1, cyclin D3, cdc2/CDK1, cdc25, and cyclin B1 were sensitive to palbociclib treatment in MCF-7/C cells, and their downregulation was significantly attenuated in MCF-7/FGFR1 cells. While the upregulation of cyclin D1 might be a mechanism more pertinent to tumors with FGFR1 and CCND1 co-amplification, our analysis of cyclin B1, cdc2/CDK1, and cdc25 suggest that FGFR1 overexpression has a broad impact on various cell cycle regulators. Consistently, recent studies showed that cyclin E amplification was linked to CDKi resistance in patient samples and cyclin E overexpression confers resistance to palbociclib in gastric cancer cells [17,20]. Since cyclin B1, cdc2/CDK1, and cdc25 are mainly involved in mitotic entry [41,42,43], their downregulation in palbociclib-treated MCF-7/C cells and attenuated changes in the MCF-7/FGFR1 cells suggests that the activation of cyclins/CDKs in G2/M transition is also a mechanism that may contribute to FGFR1-induced palbociclib resistance. The association between FGFR1 deregulation-induced CDKi resistance and the upregulation of non-cyclin D/CDK4/6 cell cycle regulators, such as cyclin B1, cdc2/CDK1, and cdc25, as well as the associated molecular interactions, warrants further investigation.

CSCs play a critical role in cancer development, metastasis, and drug resistance [44]. Aberrant FGFR signaling has been associated with CSC promotion in breast cancer development and therapeutic resistance [22,30]. We previously reported that use of FGFR inhibitor effectively impedes the stemness of breast cancer cells and mammary epithelial cells [39]. However, most previous studies on CDKi resistance focused on cell proliferation and tumor growth as the readouts [10,15]. The role of CSC deregulation in FGFR-associated palbociclib resistance has rarely been reported. In this study, we demonstrate that FGFR1 overexpression significantly attenuated the palbociclib-induced inhibition of CSC stemness of breast cancer cells, as indicated by the anchorage-independent growth and tumorsphere assays that detect CSC self-renewal. These data suggest that the possible contribution of CSC promotion to FGFR1 overexpression-induced palbociclib resistance. Importantly, given the association between enhanced stemness and FGFR1-induced palbociclib resistance, our results also suggest that assessment of various strategies targeting FGFR deregulation-associated CDKi resistance should include biomarkers that can evaluate CSC stemness in tumor treatment. In the understanding of CSC promotion in FGFR1-induced palbociclib responses, we found that the levels of total and phospho-LRP6, a transmembrane receptor involved in Wnt signal transduction, were elevated in FGFR1-overexpressing cells [37], suggesting a potential interaction between the FGFR1 and Wnt/LRP5/6/FZD complexes. Since the causal relation between FGFR1-induced CSC promotion and palbociclib resistance requires further evidence, the detailed mechanism of FGFR1, associated CSC regulation in CDKi resistance, will be followed in future studies. Further investigation may facilitate the development of CSC targeting strategies for overcoming refractory tumors with FGFR-associated CDKi resistance.

Our results indicate that FGFR1 overexpression induces the strong upregulation of ER mediated transcription, which may contribute to palbociclib resistance. Data from ERE-luciferase assays and *PS2* mRNA levels suggest that palbociclib may function as an ER agonist in MCF-7/FGFR1 cells (Figure 4). This intriguing observation could be connected to palbociclib-associated activation of Akt and ERK1/2 (Figure 4C), which needs further studies. The mRNA levels of ER target genes (*MYC, E2F1, PS2*) in palbociclib-treated MCF-7/FGFR1 cells were generally higher than the control cells, also indicating an upregulated ER signaling in FGFR1-overexpressing cells. In context with our results using PI3K/Akt targeting LY294002, MAPK/Erk targeting PD98059, and ER targeting fulvestrant/ICI 182,780 (Figure 7), FGFR1-induced ER signaling was associated with ER phosphorylation/activation in these cells. Since the activation of ER signaling may induce the expression of growth factors and the ligands that enhance RTK-Akt/ERK1/2 signaling [38], the activation of ER-RTK crosstalk in MCF-7/FGFR1 cells may be an important mechanism that contributes to palbociclib resistance. Although ER-RTK crosstalk is a classic topic in growth factor-induced tumor promotion, the results from our experiments are implicated to palbociclib resistance in multiple ways: (1) in context with FGFRs/FGFs induced regulation of ER signaling [45], our data supports ER activation as a fundamental mechanism involved in palbociclib resistance. Palbociclib alone induced the activation of ERE-mediated transcription in FGFR1-overexpressing cells, which is an additional factor that contributes to ER activation. (2) Since ER-RTK crosstalk is also involved in aberrant signaling of other growth factors, FGFR1-induced ER-RTK crosstalk and palbociclib resistance may suggest CDKi resistance to cancers, with the deregulation of other growth factors/receptors, such as IGFIR. (3) Given that ER signaling regulates a number of critical pathways in tumor growth/development, genome wide analysis of ER target genes in palbociclib-treated MCF-7/C and MCF-7/FGFR1 cells may identify more mediators of the resistance. (4) Although combination of CDKis with endocrine therapy, such as fulvestrant, is a standard-of-care treatment, stronger activation of ER signaling in cancer cells with FGFR deregulation may require additional regimens to keep ER-RTK crosstalk under check for effective management of CDKi resistance in breast cancer with FGFR deregulation.

We combined AZD4547 with palbociclib to overcome FGFR1 overexpression-associated drug resistance. Data from CI analysis and clonogenic assays clearly demonstrated the synergistic effect of the combination. In particular, the combination resulted in dramatically enhanced inhibition of cell invasion, anchorage-independent growth, and CSC self-renewal/secondary tumorsphere formation. These results demonstrate the critical role of CSC stemness promotion in FGFR1-induced palbociclib resistance and highlight the efficacy of combining palbociclib with FGFR-targeting agents on CSC stemness, as well. Use of triple combination, including CDKi, endocrine therapy, and FGFR-targeting agents, appears to be necessary for the improvement of clinical outcomes of CDKi resistant breast cancer with FGFR deregulation. Recently, the combination of FGFR-targeting erdafitinib with palbociclib and fulvestrant achieved impressive results in PDX models [4]. Given the increasing options in FGFR-targeting agents, our data suggest that a combination of the agents that may achieve maximal inhibition of CSC stemness should be tested. Moreover, since activated PI3K/Akt and MAPK/ERK1/2 signaling plays a critical role in FGFR-associated therapeutic resistance [46], a combination with agents targeting these pathways to the standard CDKi-endocrine therapy may also be effective alternatives. As mentioned earlier, including CSC biomarkers in preclinical studies and clinical trials, for testing the combinations, is of great significance.

In summary, using isogenic cell line models of luminal A breast cancer, we demonstrated that FGFR1 overexpression resulted in palbociclib resistance, which was associated with enhanced ER-RTK crosstalk and attenuated downregulation of G1/S regulators (cyclin D3, pRb-E2F1 axis) and cyclins/CDKs (cyclin B1, cdc2/CDK1, cdc25) that promote mitotic entry. Our findings, that FGFR1-induced CSC stemness contributes to palbociclib resistance, were associated with the upregulation of Wnt/β-catenin signaling, which underscores the significance of CSC regulation in drug resistance. Our data also support the strategy of combining FGFR-targeting agents with standard therapy to overcome resistance to palbociclib and/or other CDKis, as well as to includ CSC markers in clinical trials. Taken together, results from this study advance our understanding of FGFR deregulation-associated CDKi resistance and lay a foundation for further mechanistic studies.

## Supplementary Materials

The following are available online at https://www.mdpi.com/article/10.3390/cells10113008/s1. Table S1: Primer sequences used for qPCR; Figure S1: Relative colony numbers of MCF-7/C and MCF-7/FGFR1 cells treated with palbociclib; Figure S2: Relative invaded cells of MCF-7/C and MCF-7/FGFR1 cells treated with palbociclib in Transwell invasion assays; Figure S3: Relative 3D colony numbers of palbociclib-induced inhibition of 3D colony formation; Figure S4: The ratios of primary (A) and secondary (B) tumorspheres of palbociclib treated over corresponding untreated groups of MCF-7/C and MCF-7/FGFR1 cells; Figure S5: Relative colony numbers of T47D/C and T47D/FGFR1 cells treated with palbociclib.
