# Supplementary material for: FGFR1 Overexpression Induces Cancer Cell Stemness and Enhanced Akt/Erk-ER Signaling to Promote Palbociclib Resistance in Luminal A Breast Cancer Cells
Source: Cells. 2021 Nov 4;10(11):3008. doi: 10.3390/cells10113008 (PMC8616148; doi:10.3390/cells10113008)
Supplement: Supplementary file 1 [file cells-10-03008-s001.zip › cells-1329489-supplementary.pdf]

Supplementary Figures and Legends

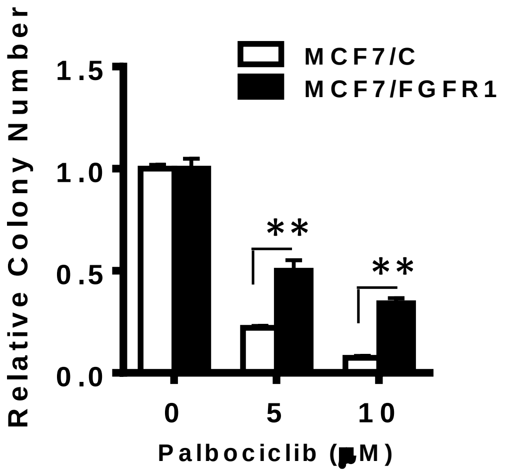

Supplementary Figure S1: Relative colony numbers of MCF-7/C and MCF-7/FGFR1 cells treated with palbociclib. The cells were treated with palbociclib (0, 5, 10 μM) for 2 weeks. The colonies were stained with crystal violet and quantified. The colony numbers presented in Figure 1C were transformed into ratios of colony numbers of drug treated cells over corresponding untreated cells (** $p < 0.01$).

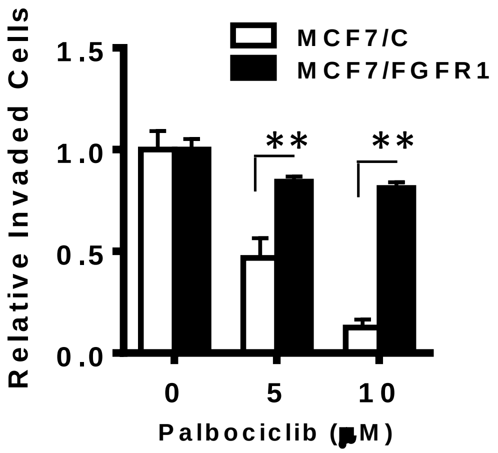

Supplementary Figure S2: Relative invaded cells of MCF-7/C and MCF-7/FGFR1 cells treated with palbociclib in Transwell invasion assays. The paired cell lines were treated with palbociclib (0, 5, 10 μM) for 30 hours in invasion chambers. The cells that invaded through the Transwell were stained, counted and analyzed. The invaded cells presented Figure 1D were transformed into ratios of invaded cells of drug treated groups over corresponding untreated groups. Data in triplicate were statistically analyzed with Student's *t*-test (** $p < 0.01$).

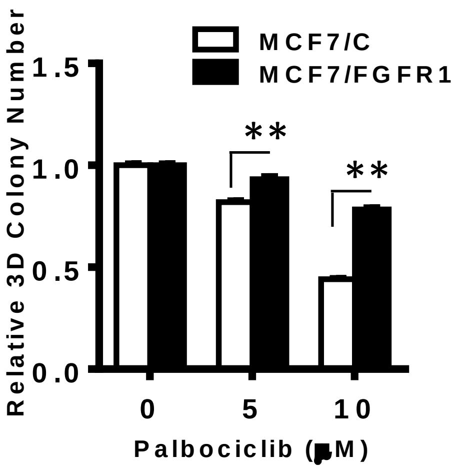

Supplementary Figure S3: Relative 3D colony numbers of palbociclib-induced inhibition of 3D colony formation. MCF-7/C and MCF-7/FGFR1 cells cultured in matrigel were treated with palbociclib (0, 5, 10 μM) for 7 days followed by colony imaging and analysis. The 3D colony numbers presented in Figure 3B were transformed to ratios of colony numbers in drug treated groups over corresponding untreated cells. Data in triplicate were analyzed with Student *t* test (** $p < 0.01$).

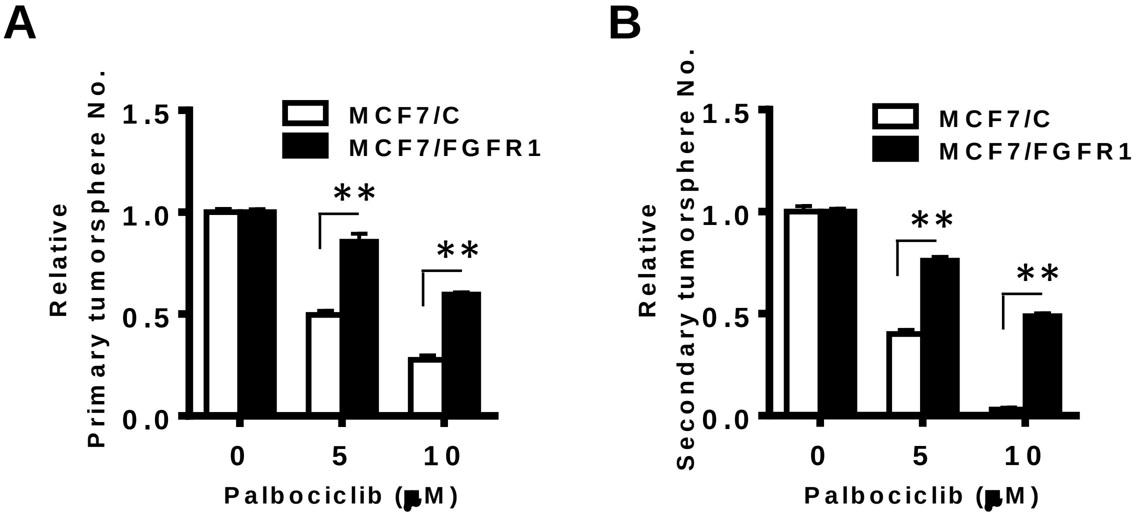

Supplementary Figure S4: The ratios of primary (A) and secondary (B) tumorspheres of palbociclib treated over corresponding untreated groups of MCF-7/C and MCF-7/FGFR1 cells. Tumorsphere assays were performed as in methods. Single cell suspensions harvested from corresponding primary spheres were then replated for secondary sphere formation (B) and analyzed. Sphere numbers presented in Figure 3B,C were transformed into ratio values of sphere formation in S5 A & B, respectively. Data in triplicate were analyzed by Student's t-test (** $p < 0.01$).

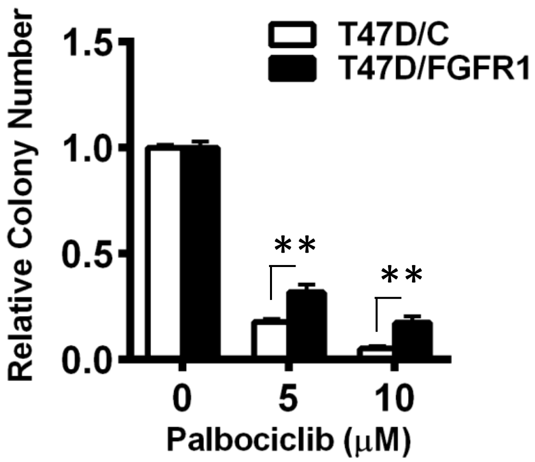

Supplementary Figure S5: Relative colony numbers of T47D/C and T47D/FGFR1 cells treated with palbociclib. The cells were treated with palbociclib (0, 5, 10 μM) for 2 weeks. The colonies were stained with crystal violet and quantified. The colony numbers presented in Figure 8D were transformed into ratios of colony numbers of drug treated cells over corresponding untreated cells. (** $p < 0.01$).

Supplementary Table S1: Primer sequences used for qPCR

Protein name	Gene name	Primer Sequences
ERα	ESR1	F: 5'-CAGGATCTCTAGCCAGGCAC-3'<br>R: 5'-ATGATCAACTGGGCGAAGAG-3'
ERβ	ESR2	F: 5'-ACCAAAGCATCGGTCACG-3'<br>R: 5'-CATGATCCTGCTCAATTCCA-3'
c-Myc	MYC	F: 5'-GGGCGCTTTGCACTGG-3'<br>R: 5'-CAGAGTAGCCTCCCCGC-3'
E2F1	E2F1	F: 5'-CATCCAGCTCATTGCCAAGAAG-3'<br>R: 5'-GATCCCACCTACGGTCTCCTCA-3'
PS2	PS2	F: 5'-ACCGCTATGTCTGTAGTGGG-3'<br>R: 5'-CGCTCCGTATTTGAGGGTCAG-3'
FGF2	FGF2	F: 5'-AGCCAGGTAACGGTTAGCAC-3'<br>R: 5'-GGAGAAGAGCGACCCTCAC-3'
GAPDH	GAPDH	F: 5'-AATGAAGGGGTCATTGATGG-3'<br>R: 5'-AAGGTGAAGGTCGGAGTCAA-3'
